# Giant cell granuloma of the maxilla. Global management, review of literature and case report

**DOI:** 10.4317/jced.50701

**Published:** 2012-04-01

**Authors:** Isidoro Rubio-Correa, Damián Manzano-Solo de Zaldívar, Raúl González-García, Luís Ruíz-Laza, Laura Villanueva-Alcojol, David González-Ballester, Cristina Hernández Vila, Florencio Monje-Gil

**Affiliations:** 1 Resident Surgeon, Department of Oral and Maxillofacial-Head and Neck Surgery,University Hospital Infanta Cristina, Badajoz, Spain; 2 Staff Surgeon, Department of Oral and Maxillofacial-Head and Neck Surgery, University Hospital Infanta Cristina, Badajoz, Spain; 3 Oral and Maxillofacial Surgeon. Staff Surgeon, Department of Oral and Maxillofacial-Head and Neck Surgery, University Hospital Infanta Cristina, Badajoz, Spain. Fellow ofthe Eurpoean Board of Oral and Maxillofacial Surgeons; 4 Head of Department of Oral and Maxillofacial-Head and Neck Surgery, University Hospital Infanta Cristina, Badajoz, Spain

## Abstract

Giant cell granuloma is a relatively rare benign entity but can be locally aggressive. Histologically characterized by intense proliferation of multinucleated giant cells and fibroblasts. Affects bone supported tissues. Definitive diagnosis is given by biopsy. Clinically manifest as a mass or nodule of reddish color and fleshy, occasionally ulcerated surface. They can range from asymptomatic to destructive lesions that grow quickly. It is a lesion to be considered in the differential diagnosis of osteolytic lesions affecting the maxilla or jaw. Its management passed from conservative treatment with intralesional infiltration of corticosteroids, calcitonin or interferon, to the surgical resection and reconstruction, for example with microvascular free flaps.

** Key words:**Giant cell granuloma, intralesional injection, microvascular free flap, fibula.

## Introduction

Giant cell granuloma (GCGs) was first described by Jaffe in 1953 (1) as “giant cell reparative granuloma”, but currently does not refer to him as reparative, because of its locally destructive. It is classified as peripheral if it affects the extremities and central if it develops in the midline (being the least common type). It is a relatively rare entity. Accounted for 7% of the maxillary tumors (his preferred location it´s the incisor region, and more frequently in the jaw than the maxilla) (2). It is more common in children and young adults, with a slight predominance in females (2). As etiological factors (3) have been related several factors, especially local irritants (such as extractions or poorly fitting dentures) and hormonal (4) (in fact, when we diagnose a GCGs, we should discard the coexistence of primary hyperparathyroidism, because classic brown tumors features of this disease are virtually indistinguishable from the histology of GCGs). Another theory relates to its origin it is an intraosseous vascular lesions similar to angiomas of soft tissues (5). In any case usually affects bone supported tissues.

## Case Report

**Figure 1:**
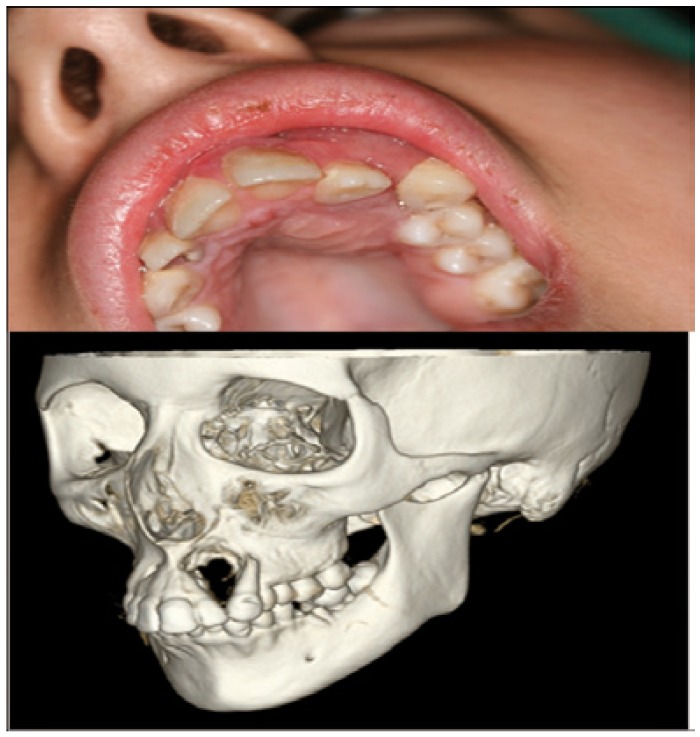
a Initial clinical view of the lesion. b View of the defect created by the lesion in 3D reconstruction CT-scan.

**Figure 2:**
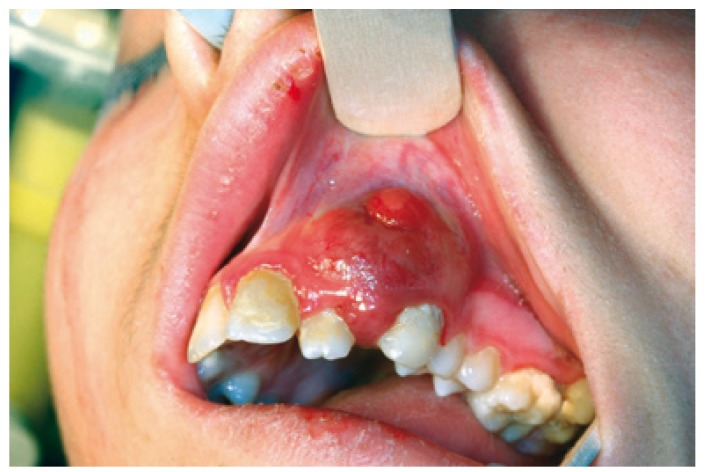
Appearance of the lesion after conservative treatment with intralesional triamcinolone.

**Figure 3:**
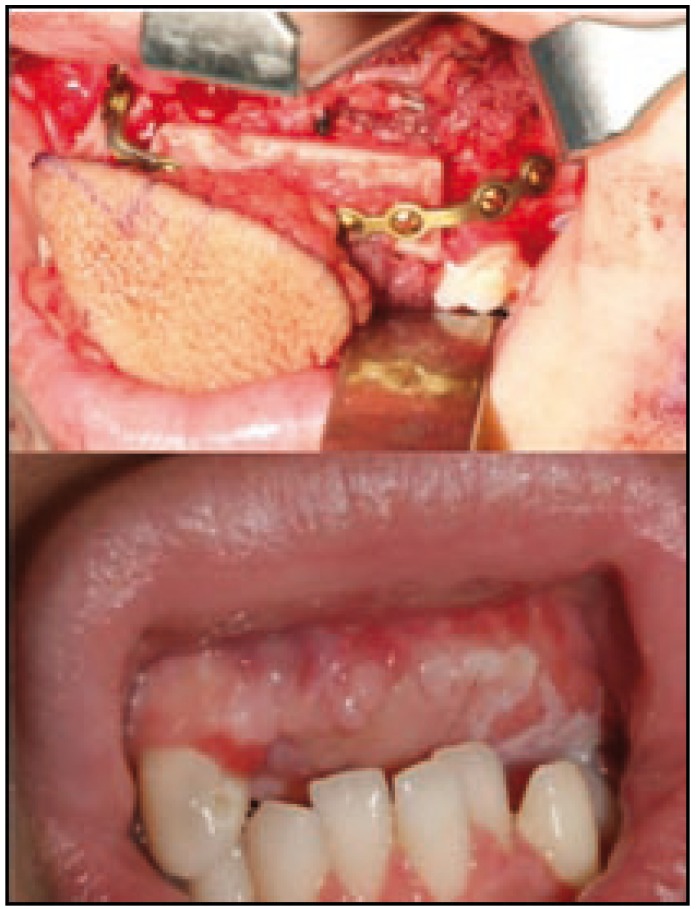
a Adaptation of microvascular fibula free flap skin paddle associated to the resultant defect after excision. b Appearance of the flap once thinned two months after the reconstructive surgery.

Female patient 16 years old with no history of interest who presents a lesion of several months, in the gingiva of the second quadrant, whose size, according to the patient, has not increased in recent weeks (Fig. 1a). It is clinically asymptomatic. On physical examination, the lesion is reddish, soft and fleshy. It causes a significant and bulging prominence, both lobby and palatal and mobility of the pieces 22 and 23. The analytical requested in its health center is completely normal. Orthopantomography shows the bone defect that coincides with lesion, pieces 18, 28, 38 and 48 included and root fragments of pieces 16 and 36, and periapical lesions in relation to pieces 36 and 37. When patient is attended in consultation, a facial, axial and coronal computed tomography (CT)-scan and 3D reconstructions are requested (Fig. 1b), which shows the defect displayed on the Orthopantomography. In Hematoxylin-eosin staining appears intense proliferation of fibroblasts and multinucleated giant cells, and it is reported as “giant cell granuloma.” Based on the age of the patient, in the absence of clinical and benign nature of the lesion, we opted for conservative treatment by six cycles of intralesional injection of triamcinolone, with the further implementation of regular radiological controls. However, due to the persistence and escalation of the lesion (Fig. 2), we completed treatment with resection of the granuloma and reconstruction of the defect with microvascular fibula free flap with skin paddle associated by anastomosis of the peroneal vessels to the facial vessels (Fig. 3a). In a second procedure, two months after reconstruction, the flap was defatted (Fig. 3b).

## Discussion

GCGs clinically manifests as a mass or nodule of reddish color (although it can sometimes be bluish) and occasionally ulcerated fleshy surface. Its range can be from asymptomatic, small and slow-growing to large and destructive lesions that grow rapidly. Imaging testing are essential, because show the true extent of GCGs and their behavior in the tissue in which it sits. Although, as a first approximation we can make use of the Ortopantomography, it is often necessary to perform CT, sometimes even three-dimensional reconstructions. Definitive diagnosis is determined by biopsy. Thus, histologically characterized (4) by the intense proliferation of multinucleated giant cells and fibroblasts, a dense vascular stroma with hemosiderin deposits. The differential diagnosis is made with pyogenic granuloma, gingival fibroma, fibrosarcoma and distant metastases of tumors, because they can resemble clinically GCGs.

As for handling, typically resorted to surgery, from simple curettage to resection of the lesion. After the surgical treatment has a high rate of recurrence (between 13-49%) (6). Currently calls for a conservative therapeutic, that prevents or diminishes the aesthetic and functional sequelae caused by standard treatments. So basically include three lines of conservative treatment. The most used is the intralesional injection of corticosteroids, with different guidelines and protocols, of which the most widespread in the literature is the weekly injection of triamcinolone (7) associated with local anesthetic for a period six weeks. Other conservative options are the use of calcitonin (8) (usually in the form of nasal spray) or interferon-α (INF-α) (4,8), both in pattern of several months, depending on the success in reducing the granuloma. Occasionally, due to the high recurrence rate or incomplete disappearance of GCGs with conservative treatment is necessary to supplement the treatment with surgical removal of the same, and if possible, reconstruction of the defect created, for example with buccal fat pad flap (9) or microvascular free flaps, such as the fibula. Whatever the method chosen, it is essential to clinical and radiological control of the patient, through revisions and imaging testing (mainly CT).

We can conclude that GCGs is a relatively rare entity of benign histologic nature but can have a very aggressive local behavior. A definitive diagnosis is reached by biopsy of the lesion. It should be present in the differential diagnosis in all expansive osteolytic lesions affecting the jaw or maxilla, such as pyogenic granuloma, gingival fibroma, fibrosarcoma and distant metastases of tumors. Its treatment includes from removal of it to the simple intralesional injection of corticosteroids or the use of calcitonin or INF-α, always with clinical and radiological control due to its high rate of recurrence. Given the failure of conservative treatment, management be should an aggressive resection and reconstruction, when it is possible. An adequate solution is microvascular fibula free flap.
